# Relationship between white matter alterations and contamination subgroup in obsessive compulsive disorder: A diffusion tensor imaging study

**DOI:** 10.1002/hbm.26282

**Published:** 2023-03-27

**Authors:** Mahvash Azarvand Damirichi, Mohammad Karimi Moridani, Seyyed Erfan Mohammadi

**Affiliations:** ^1^ Department of Biomedical Engineering, Faculty of Health, Tehran Medical Sciences Islamic Azad University Tehran Iran; ^2^ School of Biotechnology and Biomolecular Sciences, Faculty of Science University of New South Wales (UNSW) Sydney, NSW Australia

**Keywords:** contamination, diffusion tensor imaging, forceps minor, fractional anisotropy, obsessive–compulsive disorder, symptoms dimensions

## Abstract

Approximately 2%–3% of the world population suffers from obsessive–compulsive disorder (OCD). Several brain regions have been involved in the pathophysiology of OCD, but brain volumes in OCD may vary depending on specific OCD symptom dimensions. The study aims to explore how white matter structure changes in particular OCD symptom dimensions. Prior studies attempt to find the correlation between Y‐BOCS scores and OCD patients. However, in this study, we separated the contamination subgroup in OCD and compared directly to healthy control to find regions that exactly related to contamination symptoms. To evaluate structural alterations, diffusion tensor imaging was acquired from 30 OCD patients and 34 demographically matched healthy controls. Data were processed using tract‐based spatial statistics (TBSS) analysis. First, by comparing all OCD to healthy controls, significant fractional anisotropy (FA) decreased in the right anterior thalamic radiation, right corticospinal tract, and forceps minor observed. Then by comparing the contamination subgroup to healthy control, FA decreases in the forceps minor region. Consequently, forceps minor plays a central role in the pathophysiology of contamination behaviors. Finally, other subgroups were compared to healthy control and discovered that FA in the right corticospinal tract and right anterior thalamic radiation is reduced.

## Graphical abstract

In this study, we separated the contamination subgroup in obsessive–compulsive disorder and compared directly to healthy control to find regions that exactly related to contamination symptoms. Other subgroups were compared with healthy control and discovered that fractional anisotropy in the right corticospinal tract and right anterior thalamic radiation is reduced.

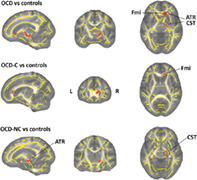

## 1 INTRODUCTION

Obsessive–compulsive disorder is a chronic mental disorder that affects 2%–3% of the world's population (Fontenelle et al., 2006). According to the World Health Organization (WHO), OCD is one of the most common disorders. OCD is characterized by obsessive thoughts and compulsive actions (Pauls et al., 2014). Obsessions are persistent, recurrent, intrusive, and unwanted thoughts, images that cause anxiety, and compulsions are repetitive behaviors or mental acts required to perform in response to obsessions governed by a rigid set of roles (Goodman, Price, Rasmussen, Mazure, & Fleischmann, 1989). Obsessions include thoughts associated with religion, sexuality, violence, or relationships, whereas compulsions involve excessive cleaning or repeated checking (Bloch et al., 2008). There is a possibility that the patient is dealing with one or both of the issues (Zaudig, 2011). OCD is associated with perception, cognition, emotional regulation, social interaction, and motor behavior, which have similarities in multiple dimensions. The heterogeneity of OCD disorder can be resolved by identifying patient subtypes using the Yale‐Brown Obsessive–Compulsive Scale (Y‐BOCS; Park et al., 2020; Van Bennekom et al., 2021). Y‐BOCS symptom checklist includes various types of compulsions such as cleaning, counting, checking, repeating rituals, ordering/arranging, and miscellaneous. There are also some obsessions in the checklist which can be mentioned, for instance, aggressive, sexual, religion, exactness, ordering, miscellaneous, somatic, symmetry, and even contamination (Goodman, Price, Rasmussen, Mazure, Fleischmann, Hill, et al., 1989). Contamination is a subtype of OCD where a person worries about contracting a disease or spreading germs (Gruner et al., 2012). People who suffer from intrusive thoughts experience severe anxiety and distress. They may engage in compulsive behaviors to relieve this stress, such as excessive washing or avoiding crowded places (Jalal et al., 2020). Researchers are able to identify factors that influence treatment outcomes by exploring symptom heterogeneity and creating more parsimonious groupings in OCD. According to Cervin et al. (2021), advanced factor analysis and network analysis techniques were used to more accurately conceptualize the nature of heterogeneous OCD symptoms using a large multinational sample of participants. It was found that previously well‐established symptom dimensions (i.e., forbidden thoughts, symmetry, contamination) contained subdimensions with a high level of theoretical relevance and face validity (Cervin et al., 2021). As with other psychiatric disorders, the therapeutic responses of OCD differ from one another, and the identification of more homogeneous subgroups may provide a solution. It is concluded that OCD has some influences on the brain, so it is possible to identify it by observing the structural changes in the whole brain (Piras et al., 2015; Soriano‐Mas et al., 2007). The structural changes are connected to the abnormal behavior of patients and cognitive dysfunction in OCD (Queiroz et al., 2021). However, the neurological origin of this disorder is still unknown. As neuroimaging techniques become more advanced, with a better spatial and temporal resolution, measurements of neurological abnormalities in psychiatric disorders are becoming more accurate. Diffusion tensor imaging (DTI), known as a noninvasive method, provides a three‐dimensional analysis of water within brain tissue (Basser et al., 1994). Measurement of the water molecules released in the human brain provides detailed information about the subtle structural changes in the brain's white matter fibers. Fractional anisotropy is the most common indicator applied to analyze water in the brain and is considered a sensitive indicator of changes in tissue microstructure. In diffusion imaging, FA is often used as a measure of white matter fiber density, axonal diameter, and myelination. FA indicates the direction of diffusion and is higher along well‐defined pathways including the corpus callosum, pyramidal tracts, and optic radiations (Basser, 1995; Mori & Zhang, 2006). FA values in the prefrontal lobe, thalamus, and cingulate gyrus have been consistently decreased in DTI studies in the last decade, hypothesizing that changes to white matter integrity may explain the development of OCD. OCD patients' responses differ sharply to treatment based on their symptoms (Calamari et al., 1999). Recent research has also shown that several neural correlates including the inferior parietal gyrus, the superior and middle temporal gyrus, and the insula are related to specific symptoms (Shan et al., 2021). Particular neural circuitry responsible for OCD is a complex disorder that is poorly understood, but dual systems in frontal‐striatal circuits may be related to OCD symptoms (Gillan & Robbins, 2014; Graybiel & Rauch, 2000).

Despite growing evidence of structural brain changes in gray matter and white matter due to OCD on brain images in recent years, a heterogeneous set of results has been represented by researchers (Zhang et al., 2021). There is a brain circuit known as the cortico‐striatal‐thalamo‐cortical (CSTC) pathway that is responsible for the execution of movements, habit formation, and reward. It has been identified that hyperactivity in the CSTC pathway contributes to OCD. The CSTC is composed of the orbitofrontal cortex, anterior cingulate cortex, basal ganglia, and thalamus. These areas have also been repeatedly shown to be affected by structural abnormalities associated with white matter abnormalities. Therefore, it is important to examine white matter changes in patients with OCD (Andrade et al., 2019; Cannistraro et al., 2007; Szeszko et al., 2005). For example, significant reductions in FA levels have been demonstrated in the cingulum, inferior fronto‐occiput, superior longitudinal fasciculus, corpus callosum, and superior longitudinal fasciculus of OCD patients (Garibotto et al., 2010). Additionally, a study of OCD patients found low FA levels in the superior frontal gyrus (SFG) and temporo‐parietal lobes (Fan, Yan, et al., 2012). Another study found lower white matter volumes in OCD patients' dorsolateral prefrontal and cingulate cortex (Lázaro et al., 2014). There are also reports that suggest significant losses in FA in the uncinate fasciculi, frontal hemispheric fibers, and Forceps minor (He et al., 2018). A combination meta‐analysis has demonstrated that OCD patients have substantial decreases in the left superior frontal gyrus, right gyrus rectus, right cerebellar hemispheric lobule, and left superior longitudinal fasciculus. A meta‐analysis of WM study results indicated that OCD patients were associated with higher FAs in the right lenticular nucleus than healthy controls, as well as lower FAs in the left insula, right cerebellum, right rectal gyrus, and left inferior parietal gyri compared with healthy controls (Niu et al., 2022). Based on subgroup analyses, there was a significant difference in FA changes between TBSS and VBA in OCD patients compared with healthy controls (Tao et al., 2022).

However, most of these studies focused on significant group differences between patients and controls. Analyses such as these failed to reflect dysfunctional neural mechanisms at the individual level and underestimated brain characteristics of specific OCD subgroups. Research on diverse aspects of the OCD subgroup is necessary to assess it better. Previous studies using whole‐brain analyses of OCD patients found that decreased FA was frequently associated with action control and OCD symptoms in the parietal, orbitofrontal, cingulate bundle, and internal capsule (Hu et al., 2020; Koch et al., 2014). The ENIGMA study on OCD brain images shows volume differences related to specific symptoms assessed by the Y‐BOCS checklist (Hazari et al., 2019). A cluster analysis examined the different symptoms among OCD patients presenting with checking and ordering symptoms and showed that their hippocampus is significantly smaller (Reess et al., 2018). There has been a disparity between the results obtained in the research conducted regarding comparing OCD patients and healthy controls. It has recently been shown in the studies discussed that certain subgroups of OCD patients exhibit special results, which are not found in other subgroups. Therefore, it can be said that one of the potential reasons for the difference in brain regions in different subgroups of OCD patients is that each subgroup of OCD patients exhibits different symptoms and experiences a variety of types of activities that may affect certain parts of the brain distinctively. According to the results obtained regarding the investigations carried out on specific subgroups in OCD, a possible explanation could be that each subgroup of OCD patients exhibits different symptoms and experiences a variety of types of activities that may affect certain parts of the brain in a distinctive manner.

Another study investigating the differences in white matter and gray matter volumes among different OCD subgroups has found that the harm/checking subgroup shows a nearly significantly decreased volume of white matter within the right superior temporal gyrus adjacent to the insula (Shan et al., 2021).

Our proposal is there should be different structural abnormalities in each of the OCD subgroups. In this study, in addition, to diagnosing OCD patients from healthy controls with DTI images, we try to compare DTI images of OCD patients in two different subgroups. We hope this study will be helpful for the accurate diagnosis of OCD patients in different subgroups.

## 2 MATERIALS AND METHODS

### 2.1 Participants

The OCD clinic at Seoul National University Hospital (SNUH, Seoul, South Korea) recruited 30 patients (female/male: 20/10; mean age: 25 ± 6.57) who met the criteria for OCD in the fourth edition of the Diagnostic and Statistical Manual of mental disorders (DSM‐IV). A structured Clinical Interview for DSM‐IV (SCID) was used to diagnose the patients. A total of 22 OCD patients were drug‐naive, and the remaining eight had been unmedicated for at least 4 weeks before inclusion. The study also includes 34 age and gender‐matched healthy controls (female/male: 23/11; mean age: 24.38 ± 3.71). All subjects were right‐handed. We removed a person in the OCD group who was 84 years old due to a significant standard deviation in age compared with other OCD patients and healthy controls and because there was no information in the demographic table about numerical assessment in any subgroups for this person and more importantly to obtain more accurate results.

### 2.2 Image acquisition

The T1‐weighted 3D magnetic resonance imaging (MRI) was performed with a 1.5T Magnetom Avanto Syngo scanner (Siemens, Erlangen, Germany) with the Time Repetition (TR) = 1160 ms, Echo Time (TE) = 4.76 ms, the flip angle of 15°, voxel size = 0.45 0.45 0.90 mm^3^, and the field of view = 350 × 263 × 350 mm^3^. DTI was also conducted on the subjects. As a result of 12 noncollinear directions with 1000 s/mm^2^ b‐factor and 10 repetitions with no diffusion weight, diffusion‐weighted images were acquired with Repetition Time (TR) = 9200 ms, Echo Time (TE) = 83 ms, field of view: 224 × 256 × 150 mm^3^ and voxel size = 2.0 × 2.0 × 2.0 mm^3^.

### 2.3 Used database

The Seoul National University Hospital Institutional review board approved the use of this data set. (Seoul, South Korea; reference number: C‐1405‐076‐581). Preprocessed DTI images in this article can be freely downloaded from Dryad (Kim, 2016). Fractional anisotropy available in this data set was extracted. The demographic table included 13 divisions from the Yale Brown checklist, which are divided into contamination/cleaning, aggressive/checking, hoarding/saving, sexual/religious, symmetry/ordering, and somatic and repeating and counting. The severity of patients' symptoms in each of these parts was scored with scores of 0 (absent symptom), 1 (symptom present but not of major concern), or 2 (prominent symptom). Among these subgroups, 50% of the patients only had contamination obsessions and cleaning compulsions, and all of the patients in this subgroup scored 2 in terms of severity. We separated it from the other subgroups to obtain more information specific to the contamination/cleaning subgroup, which covered the most comprehensive statistical number of OCD patients.

Demographics and clinical assessments in this data set do not include all the information mentioned in the recommendation note, which contains three or more indirect identifiers such as IQ, age of onset, and so forth. Although we asked for more information, we did not receive a response. The accessible demographic only includes age, gender, and psychiatric assessments.

### 2.4 Symptom assessment

The Y‐BOCS symptom checklist is a 58‐item checklist comprising 17 different subgroups of specific OCD symptoms. The Y‐BOCS symptom checklist is designed to determine how the different symptom dimensions are gathered (Rosenfeld et al., 1992). We categorize the participants with OCD into two subgroups. The first subgroup of OCD patients has contamination in our data as OCD‐Contamination (OCD‐C) (people that 2 numerical assessment in contamination) and the second subgroup of OCD patients who are not obsessed with contamination as OCD‐None Contamination (OCD‐NC) (people that 0 numerical assessment in contamination). Except for one patient with OCD, all of the participants in the contamination subgroup had 2 numerical assessments, indicating contamination is a prominent symptom in this particular subgroup. To obtain more accurate results regarding the examination of the contamination subgroup, one individual who had a 1 numerical assessment in contamination has been removed.

The characteristics of the sample used in this study are shown in Table 1.

**TABLE 1:** Sample characteristics

Variables	OCD (*N* = 28)			Significance	
	OCD‐C (*N* = 14)	OCD‐NC (*N* = 14)	HC (*N* = 34)	*t*‐Value	*p*‐Value
Age, mean ± *SD* (years)	27.14 ± 7.67	23.78 ± 5.65	24.38 ± 3.71	1.285	0.203
Gender (male|female)	8|6	10|4	23|11	−0.296	0.769

Symptom dimensions	N	%	Gender (male|female)		
Contamination	14	50	8|6		
Cleaning	12	43	9|3		
Aggressive	4	14	2|2		
Checking	7	25	6|1		
Saving	5	18	4|1		
Sexual	1	4	0|1		
Religious	1	4	1|0		
Symmetry	0	0	0|0		
Ordering	0	0	0|0		

*Note*: Paired sample test for group differences between the OCD and healthy controls are given with *t*‐value and *p*‐value.

Abbreviations: HC, healthy controls; OCD‐C, obsessive–compulsive patients with contamination symptom; OCD‐NC, obsessive–compulsive patients without contamination symptom; Y‐BOCS, Yale‐Brown Obsessive–Compulsive Scale.

### 2.5 Statistical analysis

#### 2.5.1 Diffusion tensor imaging analysis

**FIGURE 1:**
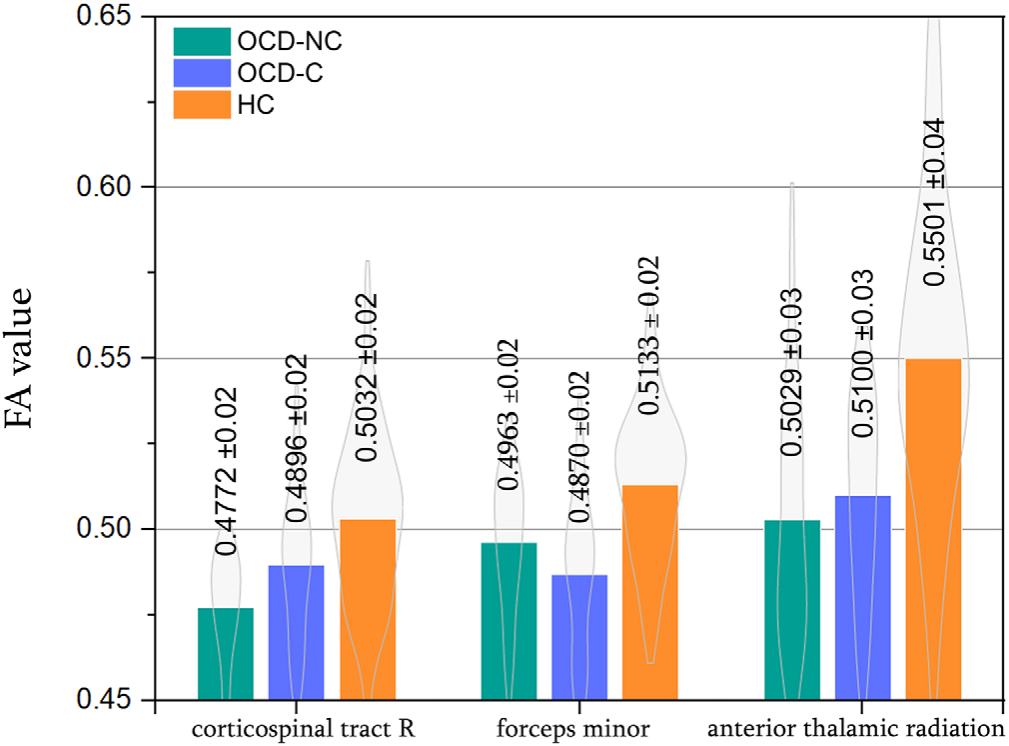
Bar plots of mean fractional anisotropy in OCD‐C, OCD‐NC, and controls in extracted areas

Analyses of the FA data are based on a voxel‐wise statistical method using tract‐based spatial statistics (Smith et al., 2006), which is a part of FSL (Smith et al., 2004). To generate a mean FA tract skeleton for each participant, TBSS applies voxel‐wise cross‐subject statistics to all FA data. The TBSS focuses on the centers of all‐fiber bundles that are common to all participants, and each FA image is aligned and affined in 1 × 1 × 1 mm MNI152 space. Next, All FA images are averaged to create a mean FA image. The mean FA is entered into the tract skeleton generation that contains the centers of the tracts, required to generate the tract skeletons for all groups. By filling in the skeleton with maximal FA values obtained from the nearest tract center, a skeletonized FA map is generated from the aligned FA images for every subject. Target images are aligned using affine MNI152 space. A two‐sample unpaired *t*‐test is used to assess differences between the OCD groups (OCD, OCD‐C, OCD‐NC) and control group with voxel‐wise cross‐grouping in all voxels with an FA of ≥0.20. The *t* statistics maps are generated using the Monte Carlo permutation test (1000 permutations). *T* statistical maps between the OCD group and the healthy controls are calculated using thresholds for statistical images with a *p* < .05 and family‐wise error correction for multiple voxel comparisons. After that, we get cluster and peak information from randomizing for each of the previous comparisons and extract significant *p*‐value areas in the MNI152 standard space. The areas in all FA images are obtained then we extract the volumes that are shown in Figure 1 for future analysis. Finally, we use Mango software to view the results and locate the differences in brain regions' coordination.

#### 2.5.2 Correlation analysis

Statistical analysis is carried out using the Statistical Package for the Social Sciences (SPSS; version 26.0; George & Mallery, 2019). The age differences are assessed using the independent samples *t*‐test. In order to investigate the further relationships between structural brain changes and symptoms, we perform a correlation analysis of all the following extracted regions with the age of OCD‐C and OCD‐NC subgroups and select all the results with *r*‐value >0.03.

## 3 RESULTS

### 3.1 Diffusion tensor imaging data

**FIGURE 2:**
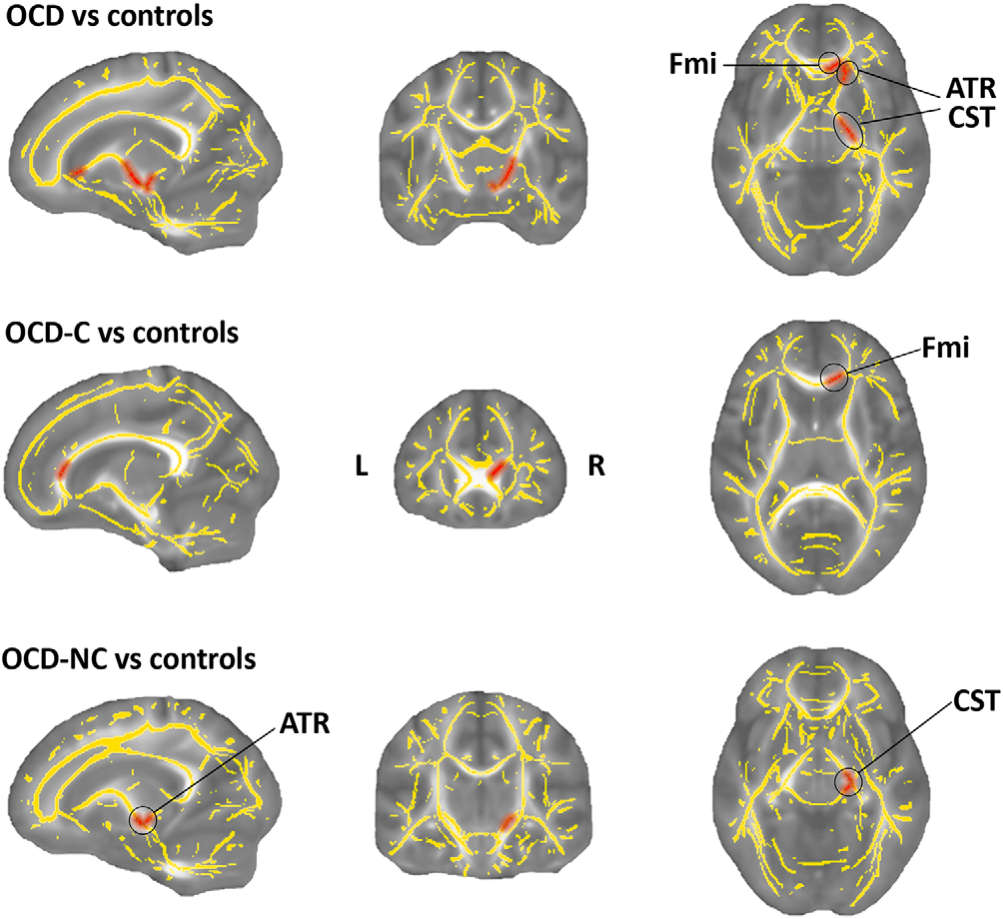
Differences in fractional anisotropy (FA) between OCD, OCD‐C, OCD‐NC, and controls. The mean FA skeleton across all subjects is shown in yellow over the FMRIB58_FA_1mm template

Comparison DTI images of OCD patients with healthy controls show that OCD patients have FA deficiency in right anterior thalamic radiation, right corticospinal tract, and forceps minor areas with a *p* < .02. Also, by comparing the contamination subgroup of OCD patients with healthy controls at *p* < .02, it is observed that OCD‐C has less FA than healthy controls in the forceps minor region. Finally, by comparing the OCD‐NC subgroup with healthy controls, it is concluded that FA in the right corticospinal tract and right anterior thalamic radiation is reduced. The coordinates of all the extracted areas along with the *t*‐value and *p*‐value, are reported in Table 2. As shown in Figure 2, separate regions were extracted by comparing each of the subgroups with healthy controls. The total of these regions was obtained by comparing OCD patients with healthy controls. Also, in all comparisons of OCD patients, lower FA levels were observed than healthy controls.

**TABLE 2:** White matter regions of lower FA in OCD‐C, OCD‐NC, and OCD compared with controls

Index	Tract label	MNI‐space				*p*‐Value
		x	y	z	*t*‐Value	FWE corrected
OCD vs. controls						
1	Right anterior thalamic radiation	10	−13	−13	3.054	0.015
2	Right corticospinal tract	15	−21	−11	2.745	0.015
3	Forceps minor	13	28	−5	2.756	0.015
OCD‐C vs. controls						
4	Forceps minor	8	29	7	3.133	0.022
OCD‐NC vs. controls						
5	Right corticospinal tract	18	−20	−9	3.252	0.018
6	Right anterior thalamic radiation	10	−13	−11	2.670	0.020

Abbreviations: FA, fractional anisotropy; FWE, family‐wise error; MNI, Montreal Neurological Institute; OCD‐C, obsessive–compulsive patients with contamination symptom; OCD‐NC, obsessive–compulsive patients without contamination symptom.

### 3.2 Clinical data

**FIGURE 3:**
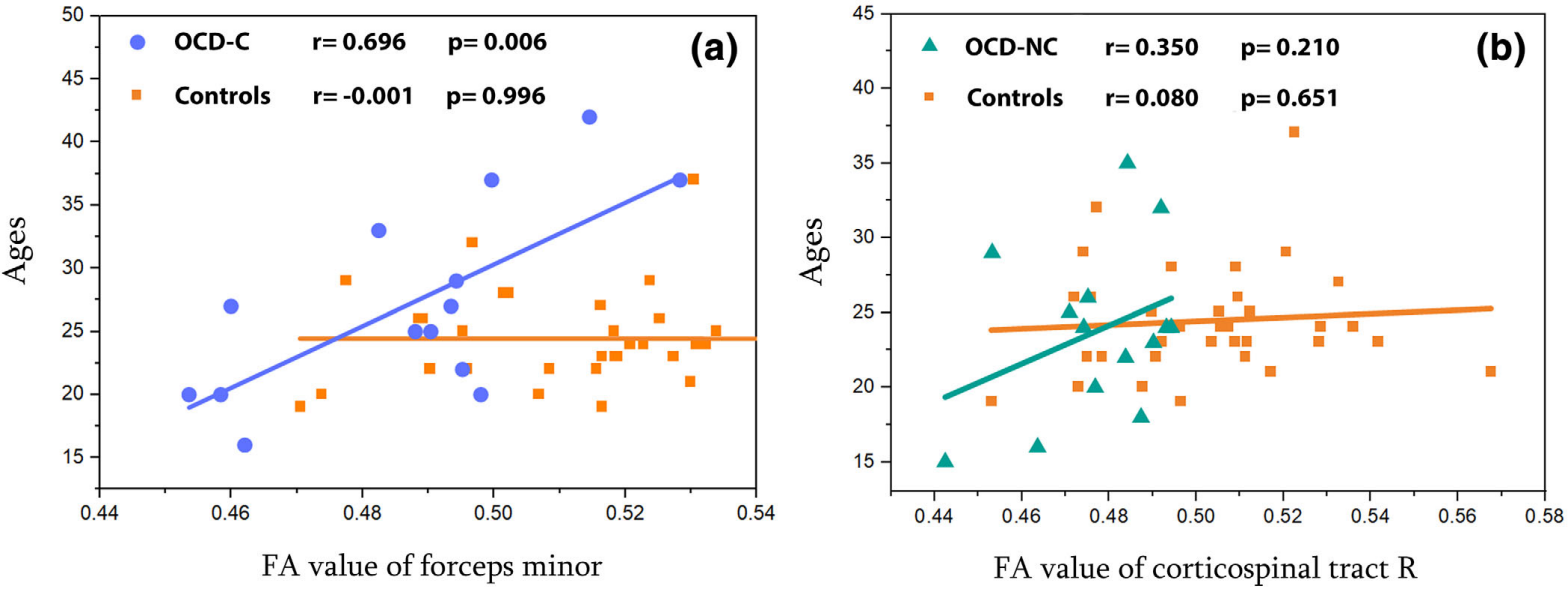
Scatterplots between fractional anisotropy (FA) values and ages of groups. (a) Correlation between FA values of the forceps minor and ages in OCD‐C and controls. (b) Correlation between FA values of the corticospinal tract R and ages in OCD‐NC and controls

In the correlation which is performed in the analysis of all extracted regions with the age of OCD‐C and OCD‐NC subgroups, OCD‐C (*r* = 0.696, *p* = .006) has a strong correlation with age in the forceps minor area. OCD‐NC also has a correlation (*r* = 0.350, *p* = .219) with age in right corticospinal tract area. The following scatter plots are displayed in Figure 3. As shown in Figure 3, no relationship was observed in healthy controls with increasing age and FA levels, but in the studied subgroups, FA level also increases with age. No significant results are obtained from other comparisons.

## 4 DISCUSSION

This study compares DTI images of OCD patients with healthy controls using the TBSS technique. We find that OCD patients had lower FA levels in right anterior thalamic radiation, right corticospinal tract, and forceps minor than healthy controls. Anterior thalamic radiation is involved in executive functions and behavior planning, which can be a reason for compulsion and obsessions. This part of the brain is one of the main fibers in fronto‐thalamic circuitry that is related to the symptoms of OCD. These results suggest a researchers have also found that pairing of structural and metabolic changes in anterior thalamic radiation, which may act as a multifaceted biomarker in the pathogenesis of OCD (Wang et al., 2018). Our findings confirm the results of the research of Haber et al. (2021) and Baldermann et al. (2021). It has also been mentioned in some recent articles that the volume of gray matter in these areas is lower in OCD patients than in healthy controls (Lv et al., 2021), but there are contradictory findings in this regard. As an example, Yoo et al. found that FA increased primarily in drug‐naive OCD patients in the thalamic radiation‐containing areas of the limb, retrolenticular parts of the internal capsule, and superolateral areas of the caudate, which were penetrated by the posterior, superior, and anterior thalamic radiations (Yoo et al., 2007). Our idea for this contradiction is that each subgroup of OCD has different symptoms, which cause different actions or thoughts, so the reason for this contradiction may be the existence of different subgroups in the statistical population of OCD patients available in research. Therefore, we have separated the contamination subgroup of OCD patients, which accounted for 50% of the population in our data. We have compared this subgroup with healthy controls and found that the contamination subgroup has less FA in forceps minor than healthy controls. Forceps minor, also known as the anterior forceps, is a white matter fiber bundle that connects the lateral and medial surfaces of the frontal lobes and crosses the midline via the genu of the corpus callosum (Gobbi et al., 2014; Riverol et al., 2009). In a recent study by Dikmeer et al. on OCD patients and their unaffected siblings, it has been inferred that OCD patients has lower FA in forceps minor, inferior fronto‐occipital fasciculus, anterior thalamic radiation, and cingulum than healthy controls (Dikmeer et al., 2021). By examining their data in their participant clinical characteristics table, we have found that OCD patients in the contamination/cleaning and aggressiveness subgroups have significantly higher scores on the Yale‐Brown checklist than the other subgroups. According to our findings, the lack of FA in forceps minor, which is shown in a comparison between OCD patients and healthy controls, is due to the predominance of the contamination subgroup according to their data.

Several documented functional and structural abnormalities of the medial and lateral frontal cortices in OCD are attributed to altered forceps minor integrity, which can result in impaired interhemispheric communication across frontal cortices. This may include those, which indicate lateralized functional abnormalities in OCD. There was an inverse correlation between FA in forceps minor and the severity of symptoms in the OCD contamination subgroup. Previous research has also demonstrated that attention‐deficit‐hyperactivity disorder (ADHD) patients exhibit a significantly lower FA in forceps minor than healthy controls and that the intensity of ADHD is also negatively associated with FA in forceps minor. Frontal cortical dysfunction that supports inhibitory control processes is not the only reason for the inability to inhibit intrusive thoughts, but also a lack of interhemispheric communication between those cortices, which may lead to compensatory over the engagement of right (or left) lateralized frontal regions on tasks requiring control processes. For example, the findings of the study by Mamiya et al. On “Predicting executive function skills in young bilingual adults” describe how the right thalamic and forceps minor may serve as attention control centers that allow bilinguals to attend to targeted presentations in their first or second languages as effectively as possible. Those participants with a higher ability to use both languages had a more significant FA in the right thalamus and forceps minor. Because of our findings, lower FA in the OCD contamination subgroup may be associated with an inability to inhibit intrusive thoughts.

We have also compared the OCD‐NC subgroup with healthy controls and found that the OCD‐NC has less FA in the corticospinal tract than the healthy controls. We have examined the amount of FA in this extracted area in all data and discovered that, according to Figure 1, the contamination subgroup in OCD has more FA in the corticospinal tract than the other OCD subgroups. A primary motor cortex sends signals to the spinal cord, which then passes on to the muscles that control the arms, legs, and body through the corticospinal tract. Consequently, this tract is responsible for the conscious movements of the muscles (Martin, 2005). According to the scores obtained from the Y‐BOCS test of OCD patients, approximately all subjects have the same scores in the contamination and cleaning subgroups. Compulsive cleaning is often related to fears of contamination (Rajkumar, 2020). Therefore, increased FA in the corticospinal tract in the contamination subgroup can be a reason for excessive cleaning habits in this subgroup. Furthermore, no significant differences were found between OCD‐C and OCD‐NC. As a possible explanation for the lack of significant differences between OCD‐C and OCD‐NC, it seems likely that one of the factors may be the relatively small number of participants in these groups.

So far, studies have shown that various FA values have been obtained from different brain regions (Fan, Luo, et al., 2012; Hartmann et al., 2016; Li et al., 2011; Yoo et al., 2007; Zhou et al., 2018). All in all, it can be concluded from our results that the heterogeneous FA regions extracted by comparing OCD patients with healthy controls possibly depend on the number of OCD subgroups available in each data set.

We have also examined the correlation between the extracted regions and the segmented groups and discovered a direct relationship between age and FA increase. In other words, by aging the FA increases in the OCD‐C and OCD‐NC subgroups in the brain's forceps minor and corticospinal tract R regions. On the contrary, no significant changes can be seen in healthy controls estimated FA in those regions. So, according to a comparison made between healthy controls and the contamination subgroup of OCD, it can be concluded that OCD‐C has less FA in the forceps minor region than healthy controls. These results represent that the amount of FA in forceps minor of older people in the OCD‐C subgroup is getting close to FA in healthy controls. Also, in the cortico‐spinal tract R, OCD‐NC has the same result. There may be several reasons for this phenomenon. For instance, it is necessary to mention that the subgroup's patients are aged between 16 and 42, with an average of 27.14 years old. This stage is an extremely crucial period in people's lives. The participants' mental conditions according to their age and lifestyle are other notable factors that may interfere with the results. For example, the situation they experience during marriage ages or the stage of their life when they are going to live and prosper independently are crucially important. Since our data was collected in Korea, the average age for women and men is 25–29 (Ma et al., 2014). Also, the age when women possibly give birth to their first child is on average 32.2 (55) (The World Factbook, n.d.). This means that at these ages, they may be very busy dealing with their personal issues, which can be a good distraction for them to lower their obsessions.

During the second decade of life, certain brain regions become more interconnected and myelin covers the main nerve fibers of the brain. As a result of this process, white matter in the brain increases, reaching its peak around age 40. Increasing white matter in the brain indicates increased communication between regions of the brain that are relatively distant from one another. During childhood and adolescence, most brain networks are regionally organized, and nearby areas cooperate in order to perform cognitive functions. As an adult, this organization is widely distributed throughout the brain. Furthermore, distant regions are able to communicate with each other and form a cooperative system. A significant amount of white matter increases in the frontal cortex during this period. Several cognitive functions, such as planning and decision making, are managed by this area. When a person reaches the age of 20–40, their brain develops a stronger capacity for cognitive control. In consequence, another explanation for the increase in FA in OCD patients between the ages of 20 and 40 years, and the FA level being close to healthy controls, could be the increase in brain white matter at this age (Carey, 1990).

### 4.1 Limitations

The present study has several limitations. First, the sample size in our study is comparatively small, and further verification of generalizability will be necessary through a larger sample size. Second, while studies have explored the clinical symptoms of the various OCD subtypes, we have not classified all OCD patients' subgroups, such as aggression and checking, because of the small sample size for the mentioned subtypes. Third, we do not have access to all patients' clinical variables such as IQ, BDI, age of onset, and so forth. Therefore, we cannot examine the effects of these factors with the obtained results. Additionally, other methods of analyzing white matter microstructure in the brain, including voxel‐based morphometry, provide additional information about the underlying brain mechanisms involved in OCD. TBSS may deliver satisfactory results, but the final importance maps superimposed on the template image may conceal some defects in data or analysis. In this article, we used several templates to preprocess DTI images and then selected the template that gave us the most accurate results for preprocessing. However, more accurate methods for selecting the template are presented by Bach et al. (2014). Based on the article by Bach et al. (2014). 3T imaging is more specific to group differences in the corpus callosum and cingulum bundle than 1.5T imaging, however, the available data were 1.5T images.

In conclusion, future research requires a larger sample size of participants and various symptoms. Dimensional information derived using the symptom checklist, while useful, is not a validated approach.

## 5 CONCLUSION

This study is one of the few studies that compare healthy people with OCD patients and compare subgroups of OCD to separate the characteristics of OCD subgroups. So far, many heterogeneous results have been reported in the neuroimaging of OCD patients with healthy controls. The miscellaneous calculated results may stem from the presence and number of different OCD subgroups in previous studies' data. There is a possibility for these heterogeneous results that many factors and different behaviors are confused with the general name of OCD, which can be related to different brain regions and make OCD a complex disorder. More details can be obtained by further categorizing and researching each subgroup. Comparing OCD subgroups directly with healthy controls can be a solution to these heterogeneous results, and determining biomarkers belonging to these subgroups can be helpful to treat this disorder in a better way. Our study shows that FA in the OCD contamination subgroup is reduced in forceps minor compared with healthy controls. Comparing the rest of the OCD subgroups with healthy controls, FA is also reduced in the right corticospinal tract and right anterior thalamic radiation. Right corticospinal tract and right anterior thalamic radiation may be biomarkers of other OCD subgroups that need to be identified to prepare a better treatment plan.

## AUTHOR CONTRIBUTORS

**Mohammad Karimi Moridani:** Resources, data curation, supervision, project administration. **Mahvash Azarvand Damirchi:** Idea preparation, investigation, formal analysis, writing—original draft. **Seyyed Erfan Mohammadi:** Data curation, formal analysis, experimental design, developing figures and tables, software. All authors have contributed to review, edit and approved the final manuscript.

## CONFLICT OF INTEREST STATEMENT

The authors declare no conflict of interest.

## Data Availability

The Seoul National University Hospital Institutional review board approved the use of this dataset (Seoul, South Korea; reference number: C‐1405‐076‐581).
